# Permeability and distribution of nerve growth factor in the brain of neonatal rats by periphery venous injection in hypoxic-ischemic state

**DOI:** 10.1186/s40064-016-3594-2

**Published:** 2016-10-28

**Authors:** Wenli Zhou, Jiantao Zhang, Guangming Wang, Limian Ling, Chaoying Yan

**Affiliations:** 1 Department of Neonatology, The First Hospital of Jilin University, 71 Xinmin Street, Changchun, 130021 Jilin China; 2 Department of Colorectal and Anal Surgery, The First Hospital of Jilin University, Changchun, 130021 Jilin China; 3 Department of Neurosurgery, The First Hospital of Jilin University, Changchun, 130021 Jilin China

**Keywords:** Nerve growth factor, Hypoxic, Ischemic, Blood–brain barrier

## Abstract

**Objective:**

To investigate the permeability of β-NGF through blood–brain-barrier (BBB) in neonatal and adult rats, and the spatial distribution of β-NGF in different brain regions in hypoxic-ischemic (HI) and normal neonatal rats.

**Methods:**

To investigate the overall permeability of β-NGF through BBB, β-NGF labeled with I^125^ was injected into adult rats, neonatal rats and HI neonatal rats via tail vein. The radioactivity of brain tissue and blood was examined and analyzed 30 min after injection. Also, brain regions including the basal forebrain, frontal cortex, hippocampus, hypothalamus, cerebellum, bulbus olfactorius and hypophysis, of all the rats were dissected and radioactivity was examined to investigate the spatial specificity of NGF permeation through BBB.

**Results:**

Statistically significant results were observed in I^125^-β-NGF contents in brain tissues of adult rats group, neonatal rats group and HI neonatal rats group (P < 0.05). Compared to the HI neonatal rats’ brain with the highest I^125^-β-NGF contents, normal neonatal rats ranks the second while the adult rats were the lowest. While for the spatial specificity examination part, I^125^-β-NGF in both HI group and control group were widely distributed in basal forebrain, frontal cortex, hippocampus, cerebellum and bulbus olfactorius. But the radioactivity in frontal cortex, hippocampus and cerebellum of HI groups are statistically higher than control groups (P < 0.05).

**Conclusion:**

β-NGF can more easily penetrate the BBB of newborn rats than adult rats via peripheral venous administration and this effect can be enhanced by HI insult. Also, this HI-induced permeation of β-NGF through BBB is more obvious in frontal cortex, hippocampus and cerebellum.

## Background

Hypoxic ischemic encephalopathy, also named as HIE, is one type of brain damage caused by prolonged insufficient blood and oxygen supply (Fatemi et al. 2009). HIE in the perinatal period is one of the most important factors leading to cerebral palsy and associated disabilities in children (Fatemi et al. 2009), while cerebral palsy is one of the most costly neurologic disabilities with an incidence of 0.2% in neonates of USA and can persists for a whole life for the affected children. The options for the treatment in HIE are quite limited and most of them are only supportive therapies including seizure control, blood pressure control and etc.

Nerve growth factor is a neurotrophic factor and neuropeptide. Interestingly, studies (Cortazzo et al. 1996; Nguyen et al. 2010; Satoh et al. 1999) found that nerve growth factor (NGF) can prevent apoptosis of neuron cells including the neural crest cells (Cortazzo et al. 1996; Satoh et al. 1999) and hippocampal cells (Nguyen et al. 2010). Mechanisms include the enhancement of superoxide anion production and suppression of hydrogen peroxide, and blockage of apoptotic cascade (Cortazzo et al. 1996; Nguyen et al. 2010). Also, Lindvall et al. (1994) found that brain damage can lead to an increase of endogenous NGF, which may contribute to nerve protection effect (Fan et al. 2007). In normal physiological conditions, NGF is one of the nerve cell growth regulatory factors involved in the proliferation, maintenance and survival in the neural cells, and is maintained at a moderately low level in the brain. In spite of the protective role of endogenous NGF in hypoxic insults or other damage of brain, the endogenous NGF is still quite limited and is difficult to exert long-lasting protection from the damage (O’Driscoll and Gorman 2005; Lindvall et al. 1992).

As the development of natural model NGF and β-NGF-cDNA cloned, exogenous NGF came to be an available therapeutic agent for neurological diseases (Bowes et al. 2000; Lorigados Pedre et al. 2002). Even though NGF is only 12 kDa in its mature form, it’s still hard for NGF to penetrate through Blood–Brain-Barrier (BBB). However, as a potential nerve cell protectant, whether and when NGF can better penetrate the blood brain barrier or be absorbed by the brain tissue is still controversial. In this paper, we studied the permeability of β-NGF through BBB and examined the absorption and distribution of NGF in newborn rats with hypoxic-ischemic brain damage, neonatal rats and adult rats in healthy condition.

## Methods

### Main reagents and instruments

β-NGF, I^125^, Sephadex G-25 medium, RSS-5100 portable digital oxygen meter, FT-613-automatic I^125^ radioactivity meter, Electronic scale, 70–80 days adult Wistar Rats, neonatal Wistar rats (7 days after birth). All animal experiments were approved by the Ethics Committee of the First Hospital of Jinlin University in Changchun, Jilin. All data were represented as mean ± SD.

### I^125^ labeling of β-NGF

Chloramine T method: 118.6 µg β-NGF, 100 ml chloramine T fluid (0.01 mol/L, pH 7.2, dissolved in PBS) and 2 mCi I^125^ were mixed at room temperature allowing the reaction to proceed for 3 min. Then 400 µl NaS_2_O_2_ was added into the system allowing to proceed for 1 min at room temperature to terminate the reaction. The mixture was then filtered through glucan coagulation G-25 column and the elution from equilibrium liquid was collected. 10% trichloroacetic precipitation method was used to measure the labelling efficiency. Merge labelling rate ≥83.367% elution with the specific activity at 14.0585 uCi/ug was collected and stored at 4 °C.

## The establishment of HI brain damage model

**Fig. 1:**
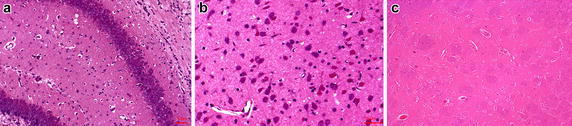
The illustration of neonatal HI rats nerve cells’ swelling with vacuoles formation (**a**), depigmentation (**a**) and hemorrhage with red blood cells leakage from the blood vessel (**b**) in hippocampus of rat. Also, the brain section of healthy neonatal rat is shown in **c**

An atmospheric oxygen cabin sized at 40 × 50 × 60 cm was made with two 2 × 2 cm holes on both sides. Electric blanket tank was used to control the environment temperature and soda lime was used to absorb moisture and CO_2_. The air within the cabin was monitored by nitrogen and oxygen conditioning test instrument (temperature is 36 ± 1 °C, oxygen concentration at 8%). Neonatal rats was kept in the cabin under hypoxic condition for 1.5 h discontinued with normoxic condition. After anoxia 30–60 min all rats manifested as cyanosis, head shaking, limb jitterring and hyperactivity. When we do HE-staining HI manifestations such as swelling, shrivel, depigmentation and bleeding can be seen (Fig. 1).

## Demographic details of each group and injection dosage

### Part 1: overall radioactivity assessment

Adult rats group: 8 rats, 70–80 days, weight to 200.4 ± 10.1 g, tail vein injection of I^125^-β-NGF 5ug/kg; normal neonatal rats group: 8 rats, born in 7 days, weight 15.8 ± 1.4 g, intravenous injection I^125^-β-NGF 5ug/kg; HI neonatal rats group: 8 rats, born in 7 days, weight 15.2 ± 1.6 g, intravenous injection I^125^-β-NGF 5ug/kg.

### Part 2: tissue specific radioactivity assessment

HI group: 16 rats, born in 7 days, weight 14.9 ± 1.7 g, hypoxia model, 8 rats to inject I^125^-β-NGF, and 8 rats to inject I^125^.

Control group: 16 rats, born in 7 days, weight 15.1 ± 1.6 g, no HI intervention, 8 rats to inject I^125^-β-NGF, and 8 rats to inject I^125^.

## Experimental design

### Part 1: overall radioactivity assessment

After the 30 min hypoxic treatment (or control treatment), adult rats group, HI neonatal group and control neonatal group were isoflurane anesthetized and euthanized by jugular vein bloodletting method. The pia mater were removed carefully followed by brain tissue separation and blood collection at the same time. The blood and brain tissue were weighed by electronic scales and the radioactivity of each rat was measured within 12 h.

### Part 2: tissue specific radioactivity assessment

After 30 min pre-treatment and all rats were isoflurane anesthetized and sacrificed, different brain parts of the rats were dissected on ice including olfactory bulb, the frontal cortex, hippocampus, thalamus region, the cerebellum, the pituitary gland. Gamma free analyzer was adopted for radioactivity measurement.

### Part 3: apoptotic analysis via TUNEL assay

After radioactivity measurement of all the brain tissues, samples were collected for 0.4% paraformaldehyde fixation for 15 min followed with paraffin-wax embedding. Slides were cut and stained with H&E staining and Terminal deoxynucleotidyl transferase (TdT) dUTP Nick-End Labeling (TUNEL) assay, as described by the protocol from the kit (MK1020, Boster, Wuhan, China).

### Statistical analysis

SPSS17.0 statistic software was used for data analysis and one-way ANOVA was used to decide the statistical significance. All experiments involved were repeated at least three times and data in this paper were shown as mean ± SD. A P value less than 0.05 is set to be statistically significant.

## Results

### Part 1: overall I^125^-β-NGF level in brain and blood of each group

**Fig. 2:**
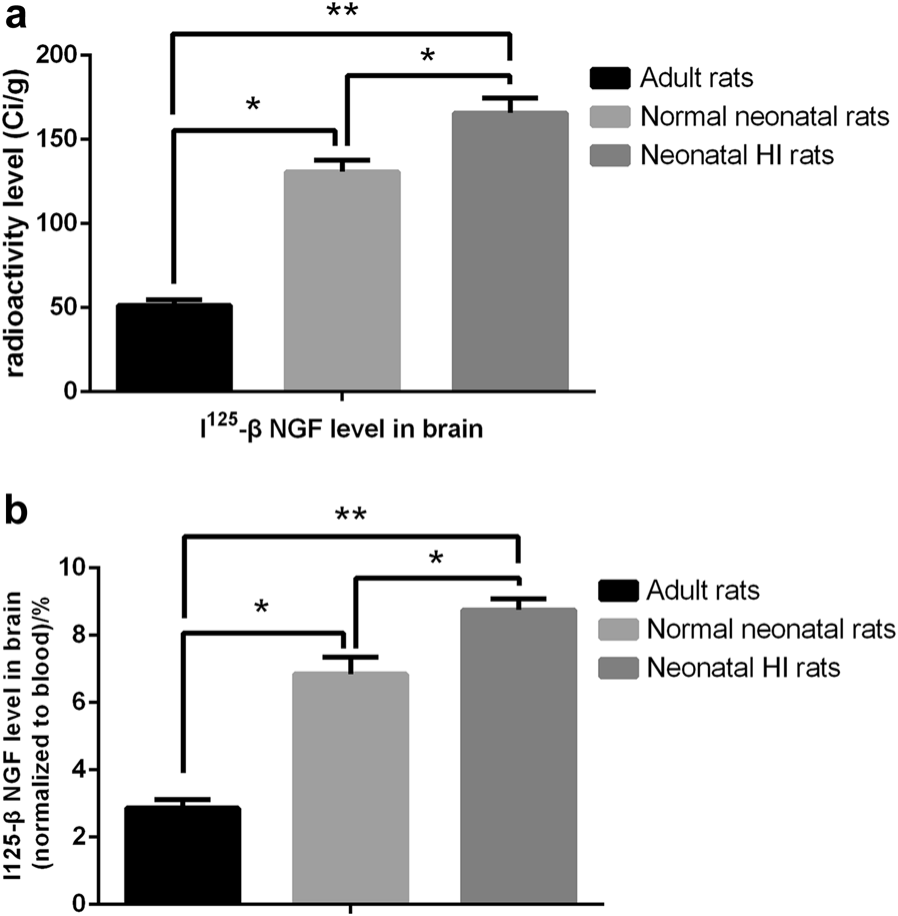
The comparison of I^125^-β-NGF level in brain via vein injection. The I^125^-β-NGF level in the brain of adult rats, normal neonatal rats and neonatal HI rats were determined quantitatively and neonatal HI rats would detain the most I^125^-β-NGF, while adult rats detain the least (**a**). Similar difference in the retention rate were found as we normalized the radioactivity of the brain to the blood between these three groups (**b**). (*P < 0.05; **P < 0.01)

Table 1 suggest there are significant difference (P < 0.05) of I^125^-β-NGF content in adult rats (51.21 ± 3.40 Ci/g), normal neonatal rats (130.74 ± 6.84 Ci/g) and neonatal HI rats (165.83 ± 8.74 Ci/g), among of which the neonatal HI group (165.83 ± 8.74 Ci/g) has the highest level of radioactivity (Fig. 2a). In the meantime, after normalization with blood I^125^-β-NGF (adult rats: 1798.61 ± 116.66 Ci/g; Normal neonatal rats: 1908.96 ± 62.71 Ci/g; Neonatal HI rats: 899.89 ± 162.96 Ci/g), brain I^125^-β-NGF of the adult rats group (2.86 ± 0.25%) is the lowest, normal neonatal rats group (6.85 ± 0.50%) take the second place, and neonatal HI rats group (8.75 ± 0.33%) is the highest (P < 0.05) (Fig. 2b).

**Table 1:** The I^125^-β NGF level in brain and blood by vein injection (Ci/g, n=8)

Group	Adult rats	Normal neonatal rats	Neonatal HI rats
Brain	51.21 ± 3.40^#^	130.74 ± 6.84**	165.83 ± 8.74*
Blood	1798.61 ± 116.66	1908.96 ± 62.71	899.89 ± 162.96
Percentage%	2.86 ± 0.25^#^	6.85 ± 0.50**	8.75 ± 0.33*

The adult rats’ brain can detain the fewest NGF at (2.86 ± 0.25)%, normal neonatal rats ranked the middle at (6.85 ± 0.50)% and neonatal HI rats can detain the most NGF at (8.75 ± 0.33)%

^#^ versus ** P < 0.05; ^#^ versus * P < 0.01; ** versus * P < 0.05

### Part 2: I^125^-β-NGF distribution in the brain of neonatal rats

**Fig. 3:**
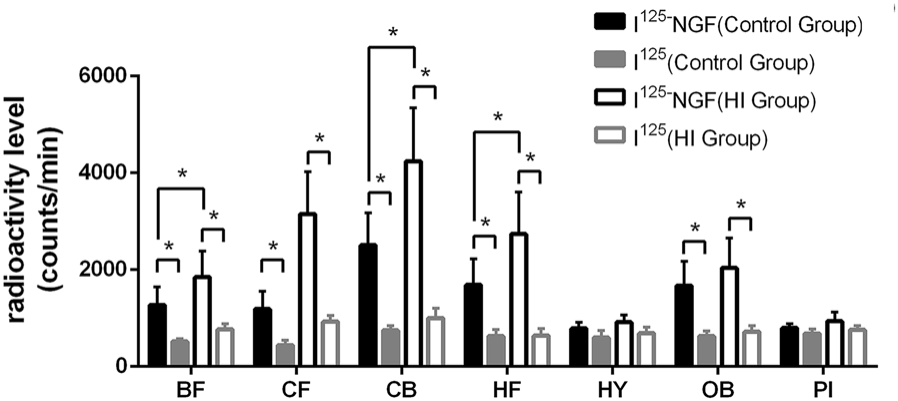
Comparison of radioactivity absorption in different brain regions. I^125^-β-NGF absorption was higher than that of I^125^ in the basal forebrain, frontal cortex, cerebellum, hippocampus, and olfactory bulb; I^125^-β-NGF in HI group absorb obviously higher than that of I^125^ in the basal forebrain, frontal cortex, cerebellum, hippocampus, and olfactory bulb. No statistically significant difference were observed in other regions of the brain (*P < 0.05)

In normal control group, I^125^-β-NGF absorption was obviously higher than that of I^125^ in the basal forebrain (1273.333 ± 376.600 vs 518.000 ± 61.454 counts/min), frontal cortex (1185.500 ± 371.714 vs 443.833 ± 101.075 counts/min), cerebellum (2505.000 ± 665.695 vs 745.000 ± 100.349 counts/min), hippocampus (1686.833 ± 534.053 vs 628.000 ± 140.274 counts/min), and olfactory bulb (1672.667 ± 500.930 vs 629.000 ± 105.284 counts/min) (P < 0.05); I^125^-β-NGF in HI group absorb obviously higher than that of I^125^ in the basal forebrain (1845.667 ± 534.705 vs 767.833 ± 116.393 counts/min), frontal cortex (3151.333 ± 872.215 vs 928.000 ± 130.545 counts/min), cerebellum (4243.833 ± 1097.127 vs 1001.000 ± 203.048 counts/min), hippocampus (2736.167 ± 873.323 vs 637.000 ± 151.060 counts/min), and olfactory bulb (2036.167 ± 620.084 vs 715.000 ± 130.026 counts/min) (P < 0.05) (Fig. 3; Table 2).

**Table 2:** Comparison of radioactivity absorption in different brain regions (counts per minute, $\bar{x}$ ± s)

Brain region	Control group		HI group	
	I^125^-NGF	I^125^	I^125^-NGF	I^125^
BF	1273.333 ± 376.600	518.000 ± 61.454	1845.667 ± 534.705	767.833 ± 116.393
CF	1185.500 ± 371.714	443.833 ± 101.075	3151.333 ± 872.215	928.000 ± 130.545
CB	2505.000 ± 665.695	745.000 ± 100.349	4243.833 ± 1097.127	1001.000 ± 203.048
HF	1686.833 ± 534.053	628.000 ± 140.274	2736.167 ± 873.323	637.000 ± 151.060
HY	789.333 ± 123.072	595.333 ± 145.446	919.000 ± 145.213	686.500 ± 131.991
OB	1672.667 ± 500.930	629.000 ± 105.284	2036.167 ± 620.084	715.000 ± 130.026
PI	796.333 ± 89.189	684.333 ± 93.520	936.500 ± 184.183	755.333 ± 94.118

I^125^-β-NGF absorption was obviously higher than that of I^125^ in the basal forebrain, frontal cortex, cerebellum, hippocampus, and olfactory bulb (P < 0.05); I^125^-β-NGF in HI group absorb obviously higher than that of I^125^ in the basal forebrain, frontal cortex, cerebellum, hippocampus, and olfactory bulb (P < 0.05)

*BF* basal forebrain, *CF* frontal cortex, *CB* cerebellum, *HF* hypocampus, *HY* hypothalamus, *OB* bulbus olfactorius, *PI* hypophysis

I^125^-β-NGF in HI group absorb obviously higher than that of I^125^-β-NGF in normal control group in the frontal cortex (3151.333 ± 872.215 vs 1185.500 ± 371.714 counts/min), cerebellum (4243.833 ± 1097.127 vs 2505.000 ± 665.695 counts/min) and hippocampus (2736.167 ± 873.323 vs 1686.833 ± 534.053 counts/min) (P < 0.05). NGF HI group absorb in the basal forebrain (1845.667 ± 534.705 vs 1273.333 ± 376.600 counts/min), hypothalamus (919.000 ± 145.213 vs 789.333 ± 123.072 counts/min), olfactory bulb (2036.167 ± 620.084 vs 1672.667 ± 500.930 counts/min) and hypophysis (936.500 ± 184.183 vs 796.333 ± 89.189 counts/min) have no obvious difference to NGF normal group in the above areas (Fig. 3, Table 2).

### Part 3: NGF supplement ameliorated the apoptosis in the hippocampus region

**Fig. 4:**
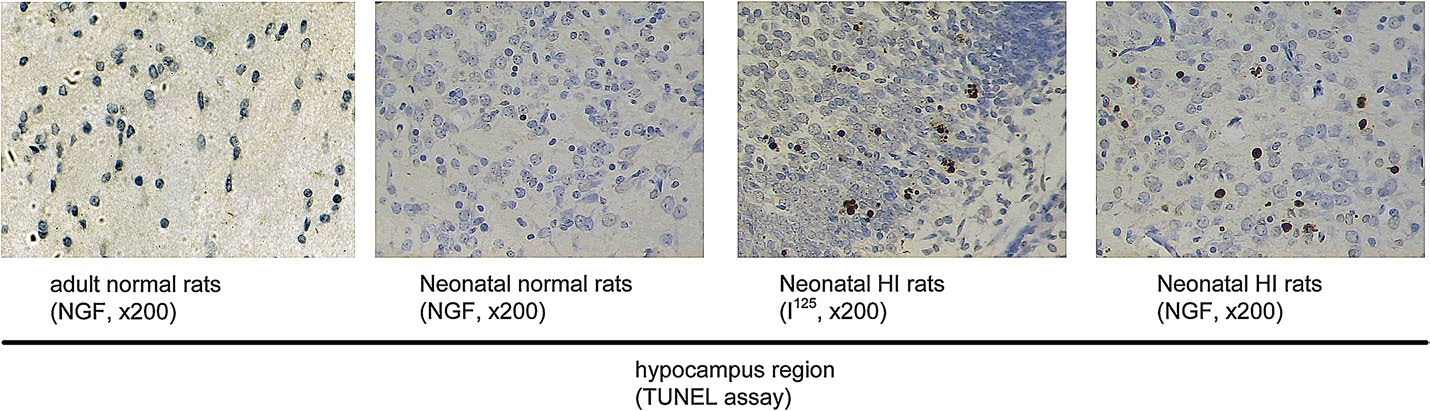
The TUNEL assay in the hippocampus region of the adult normal rats with NGF injection, the neonatal normal rats with NGF injection, the neonatal HI rats with NGF injection, neonatal HI rats with PBS injection

As shown in Fig. 4, there are much more cells undergoing apoptosis after HI insult in the brain. Also, as we apply NGF in the neonatal HI group, we found the apoptosis in the hippocampus of neonatal HI group with NGF injection is much milder compared to control group with only I^125^ injection. In addition, the injection of NGF in the adult rats group does not made any changes in the hippocampus region.

## Discussion

Up to date, consensus has been reached in the therapeutic use of NGF via peripheral vein in the repair and regeneration in central nervous system (Kamei et al. 2007; Gould et al. 2000; Ferenz et al. 2012). However, because of the uniqueness of central nervous system, the blood-cerebrospinal barrier or blood–brain barrier (BBB) makes it ineffective for most drugs entering the brain (Smith et al. 2004). At present, most of the experiments use the embedded method or lateral ventricle injection, which requires intensive staff training and the successful rate of which largely depend on the operators’ skill. Also, lateral ventricular injection would lead to cerebral edema and increase the risk of infection, therefore its clinical application is greatly limited. Before the start of this study, we made a preliminary investigation on NGF level in adult rat’s brain tissues after intravenous injection and it showed that fractional amount of NGF pass through the blood brain barrier after peripheral injection of NGF, and the peak can only be achieved 30 min after intravenous injection (data not shown).

The result indicates that, the BBB permeability of NGF in newborn rat is far better than that of adult rats after intravenous injection, especially in hypoxic state. Also, in the study of NGF’s spatial distribution in different brain regions, we noticed that the uptake of I^125^-NGF in normal group and hypoxia group was obviously higher than that of I^125^ in the basal forebrain, frontal cortex, cerebellum, hippocampus and olfactory bulb, suggesting that NGF can overtly penetrating the blood–brain barrier in these brain areas. Moreover, I^125^-NGF hypoxia group absorption is significantly higher than normal control group in the frontal cortex, hippocampus and cerebellum (P < 0.05). This finding demonstrates that the newborn rat under hypoxia condition have better permeability of blood brain barrier to NGF thus it’s might be efficacious to administer venous NGF for neonates with hypoxic ischemic encephalopathy (HIE).

The mechanism of HI-neonates brain’s high permeable BBB can be explained as follows: (1) BBB is not intact in physiological state. There are different defects on BBB in areas as median eminence, the hypothalamus endplate vascular bed, sub-dome and choroid area. NGF may go through these areas into the brain tissue. (2) Hafny found that NGF may activate the gamma transpeptidase glutamic acid and alkaline phosphatase in BBB endothelial cells, which can transform those “guardian cells” of BBB into the transfer state (el Hafny et al. 1996). (3) The BBB of newborn rat is still immature thus more permeable to substances as NGF. (4) Recently Moser et al. (2004), Calza et al. (2001) prove that NGF can act as a angiogenic factor or indirectly interact with VEGF, promoting the proliferation of brain capillary endothelial cells and selectively inducing angiogenesis after cerebral ischemia, thus making the brain vessels more active in a pathophysiological state. (5) In hypoxic condition, a large number of free radicals would damage the integrity endothelial cells and glial cells’ membrane thus damaging blood–brain barrier. Also, some special transport mechanisms (Arnett et al. 2007) in HI state as unsaturated diffusion (depending on the bond energy of hydrogen bonding), saturation transport system (for a specific carrier) and etc. would make peripheral peptide drugs pass through the BBB possible (Zhang et al. 1999). In our experiment, exogenous NGF can pass through the blood brain barrier in normal state and anoxic state, and in both circumstances it can be absorbed in Cornu Ammonis significantly. So does in the cerebral cortex, the olfactory bulb, the basal forebrain and cerebellum, the distribution of which are basically the same with endogenous NGF in physiological condition (Tian et al. 2012). However, the endogenous NGF react differently in neonatal rats with hypoxia injury as compared to adult rats in the same condition. Compared to adult rats in HI state, NGF level drops quickly after hypoxia insult in newborn hippocampus followed with delayed increase in cerebellum (Scheepens et al. 2003). Therefore, it is promising that exogenous NGF can be therapeutic agents when endogenous NGF in early stages of HI insult cannot meet the actual need of the brain, by means of which increase the survival of neurons and improve the brain function after hypoxia. The application of NGF in early stages of hypoxia can limit neuron’s injury after ischemia (Lee et al. 2008). Also, the combination therapy of mesenchymal stem cells and NGF in stroke patients might also be promising to improve the life quality of patients (Jiang et al. 2013). While for the negative results in our experiments, it is speculated that NGF’s permeation through the olfactory bulb, hypothalamus, basal forebrain and hypophysis are still limited even in HI state. Also, it still cannot be ruled out that the inspection duration of this experiments might be too short to get a statistically significant difference.

Therefore, it is still in great need to further investigate the mechanism of BBB in hypoxic-ischemic state and enhance the permeability of exogenous NGF, thus improving the prognosis of patients with hypoxic and ischemic encephalopathy as strokes.

## Conclusion

β-NGF can more easily penetrate the BBB of newborn rats than adult rats via peripheral venous administration and can be enhanced by HI insult. Also, this HI-induced influx of β-NGF through BBB is more obvious in frontal cortex, hippocampus and cerebellum.

## Abbreviations

BBB: blood-brain-barrier
HI: hypoxia-ischemia
BF: basal forebrain
CF: frontal cortex
CB: cerebellum
HF: hypocampus
HY: hypothalamus
OB: bulbus olfactorius
PI: hypophysis
